# Assisted closed-loop optimization of SSVEP-BCI efficiency

**DOI:** 10.3389/fncir.2013.00027

**Published:** 2013-02-25

**Authors:** Jacobo Fernandez-Vargas, Hanns U. Pfaff, Francisco B. Rodríguez, Pablo Varona

**Affiliations:** Grupo de Neurocomputación Biológica, Departamento de Ingeniería Informática, Escuela Politécnica Superior, Universidad Autónoma de MadridMadrid, Spain

**Keywords:** brain-computer interface, brain-machine interface, activity-dependent stimulation, resting state EEG, resting state network, individual alpha frequency, BCI illiteracy, BCI performance predictor

## Abstract

We designed a novel *assisted closed-loop optimization protocol* to improve the efficiency of brain-computer interfaces (BCI) based on steady state visually evoked potentials (SSVEP). In traditional paradigms, the control over the BCI-performance completely depends on the subjects' ability to learn from the given feedback cues. By contrast, in the proposed protocol *both* the subject and the machine share information and control over the BCI goal. Generally, the innovative *assistance* consists in the delivery of online information together with the online adaptation of BCI stimuli properties. In our case, this adaptive optimization process is realized by (1) a *closed-loop search* for the best set of *SSVEP* flicker frequencies and (2) feedback of actual *SSVEP* magnitudes to both the subject and the machine. These closed-loop interactions between subject and machine are evaluated in *real-time* by continuous measurement of their efficiencies, which are used as online criteria to adapt the BCI control parameters. The proposed protocol aims to compensate for variability in possibly unknown subjects' state and trait dimensions. In a study with *N* = 18 subjects, we found significant evidence that our protocol *outperformed* classic SSVEP-BCI control paradigms. Evidence is presented that it takes indeed into account interindividual variabilities: e.g., under the new protocol, baseline resting state EEG measures predict subjects' BCI performances. This paper illustrates the promising potential of *assisted closed-loop* protocols in BCI systems. Probably their applicability might be expanded to innovative uses, e.g., as possible new diagnostic/therapeutic tools for clinical contexts and as new paradigms for basic research.

## Introduction

The use of closed-loop interaction with biological nervous systems for observation and control purposes goes back to the beginnings of electrophysiology in the 1940s when the *voltage clamp* technique was developed (Marmont, 1949; Cole, 1955). Later on, the *dynamic clamp* technology to implement artificial membrane or synaptic conductances (Robinson and Kawai, 1993; Sharp et al., 1993) has produced many examples of successful closed-loop interactions with neural systems at the cellular and circuit levels (for reviews see Prinz et al., 2004; Goaillard and Marder, 2006; Destexhe and Bal, 2009; Economo et al., 2010).

We recently proposed a generalization of the dynamic clamp concept in electrophysiology and animal ethology to design closed-loop interactions with biological nervous systems beyond electrical stimulation and recording. In particular, we investigated in our previous work goal-driven real-time closed-loop interactions with drug microinjectors, mechanical stimulation devices and video event driven stimulators (Muniz et al., 2008, 2011; Chamorro et al., 2009, 2012). These examples illustrate that modern activity-dependent stimulation protocols can reveal dynamics otherwise hidden under traditional stimulation techniques, provide control of regular and pathological states, induce learning processes, bridge between distinct levels of analysis and lead to a further automation of experiments. In this paper, we propose the same assisted closed-loop approach described in our previous work to optimize the efficiency of steady state visually evoked potentials (SSVEP) based brain-computer interfaces (BCI) which might have a large impact for applied uses, such as computer control and biomedical or prosthetic uses, but also as novel paradigms for basic research. Generally, the innovative assistance consists in the delivery of online information with regard to the control over the given BCI goal both to the human subject and to the system, together with the online adaptation of BCI stimuli properties.

BCIs use measures of brain activity, typically real-time human EEG recordings, usually in order to interact with devices such as virtual keyboards, etc. (for recent reviews see e.g., Birbaumer, 2006; Van Gerven et al., 2009; Nicolas-Alonso and Gomez-Gil, 2012). Among the most successful BCIs are those which rely on SSVEPs, a type of event related potentials (ERPs) generated by the nervous system in response to repetitive visual stimulation (flicker) by linear superposition of transient visually evoked potentials (VEPs) (Capilla et al., 2011) up to 90 Hz (Herrmann, 2001): apart from smaller responses in higher harmonic frequencies, the brain mainly generates electrical activity at just the same fundamental frequency as its visual system is exposed to the visual flicker frequency. SSVEPs are frequently used in basic and applied research because of their relatively large magnitudes which lead to superior signal-to-noise ratios (SNRs) and make them relatively stable against artifacts as compared to other ERPs (Vialatte et al., 2010).

SSVEP-BCIs make use of the physiological property that SSVEP magnitudes can be modulated by visual-spatial selective attention (e.g., Morgan et al., 1996). Thus, SSVEP based BCIs employ multiple visual stimuli (e.g., LEDs or regions on a screen) flickering at different frequencies. Apart from these intraindividual state changes due to attention, SSVEP magnitudes further depend both on extrinsic variables as the spatial and temporal frequencies of the stimulus, and on other intrinsic intra- and interindividual dimensions of the subjects themselves (Ding et al., 2006; Lopez-Gordo et al., 2011). The optimal spatial frequency of a structured stimulus is related to individual traits such as visual acuity or age (Vialatte et al., 2010). There is also a significant difference in the magnitude of SSVEPs between flicker stimulation of the center (fovea centralis) vs. the periphery of the visual field. Environmental conditions (e.g., screen brightness and frequency, distance to the screen, etc.) also influence the performance of the BCI. Although determined by multiple factors, SSVEP magnitudes are modulated by the subjects' states of attention. Hence, online monitoring of SSVEP magnitudes elicited by arrays of multiple flickering light sources allows BCI systems to detect to which flicker source the subject is attending to at a given moment. Taken altogether, these aspects call for automated mechanisms to optimize parameters of the stimuli and of the BCI control, aiming toward flexible adaptiveness to specific individual and contextual situations of SSVEP-BCI use.

**Figure 1:**
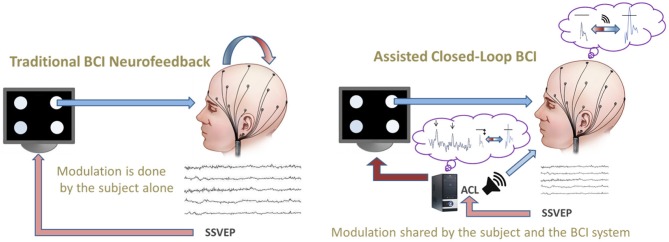
**Comparison of a *traditional* BCI neurofeedback (left) vs. the novel *assisted closed-loop* paradigm (right) which informs both the subject (about his/her brain activity in relation to the BCI goal) and the system (about the specificities of the given subject).** In our example, the assisted closed-loop provides *online* information (i) to the system about the most effective flicker frequencies and (ii) to the subject about the actual distance to the pre-defined threshold by continuous auditory feedback (loudspeaker symbol, right).

Commonly, SSVEP-BCIs use only one prefixed set of flicker frequencies, but nonetheless there are studies employing two different prefixed sets (e.g., Volosyak et al., 2009, 2011) which lead to remarkably different results. Those findings imply that BCI efficiency may crucially depend on flicker frequency selection. Following this idea, we created an assisted closed-loop adaptive algorithm to search for the best frequencies for each subject and for each particular time point/situation of use. The adaptive and informative nature of this novel online approach aims to improve the BCI efficiency as compared to traditional paradigms (see Figure 1). Firstly, this optimization process is realized by performing a real-time closed-loop search for the best set of frequencies to achieve the given BCI goal. The number of stimuli and their effectiveness with regard to the BCI goal modulate this real-time search strategy. The closed-loop search is evaluated in real-time by a continuous measurement of the actual BCI efficiency (see section “Efficiency Measures”), which is used as an online criterion to select the BCI control parameters. Secondly, the SSVEP online recording is processed, on the one hand, to an online auditory feedback to inform the subject and, on the other, is used to inform the system to select the best flicker frequencies. This shared information constitutes the assisted part of the closed-loop. The proposed protocol aims to address the problems which arise from different hardware configurations, subjects' intra- and inter-individual variabilities, e.g., in neuropsychological dimensions of executive functioning (see e.g., Funahashi, 2001) etc., and other sources of variability in experimental settings and intrinsic dimensions.

The paper is organized as follows: in section “Materials and Methods” the new assisted closed-loop system is described; in section “Results” analyses and correlates efficiency as compared with traditional BCI paradigms are presented; finally, in “Discussion” section we discuss about the generalization and applicability of the proposed novel protocol.

## Materials and methods

### Participants

A convenience non-probability sample of *N* = 18 healthy subjects from our department was used applying the exclusion criteria self-reported chronic medication/substance intake and neurological diseases as e.g., epilepsy. Our sample consisted of 6 females and 12 males with age *Mdn* = 26.00 years (25th percentile = 23.00, 75th = 35.75), range = 18–59. Subjects had a normal or corrected-to-normal vision and were right-handed. Permission of the ethics committee of Autonomous University of Madrid was obtained; all subjects participated voluntarily in the sense of an *informed consent* without receiving any incentives. Participants were informed that they could leave the experiments at any time without giving any explication.

### SSVEP BCI system

#### Stimulation device

We constructed a stimulation panel with four white color LEDs (manufacturer *Seoul Semiconductor, white lamp LED LW500AM*, ∅ 5 mm, viewing angle 100°), using a 100 Ω series resistor to the digital +5V output of the acquisition board (see below) which results in a luminous intensity output *I*_*V*_ ≈ 700 mcd for each LED.

**Figure 2:**
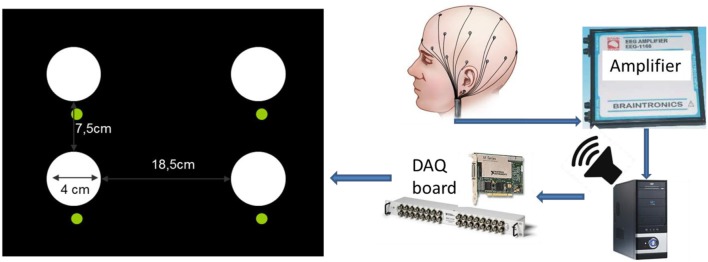
**Diagram of the *BCI flicker stimulation setup* (left) and the signal acquisition/stimulation system.** The flickering frequency was controlled by a software driving the digital output of a National Instruments data acquisition (DAQ) board (model *NI-PCI-6251*) directly connected to the white colored LEDs, generating 0/+5V *off* vs. *on* signals according to the desired flicker frequency. We verified the intended flicker frequency for each light source independently by a photodiode connected to a digital oscilloscope. Luminous intensity output is *I*_*V*_ ≈ 700 mcd for each white LED. Smaller green color standard signaling LEDs were placed below to instruct subjects where to look during the BCI task.

On a black background panel, each LED was mounted into a reflector with ∅40 mm diffuser cap carrying an outstanding non-transparent cylindrical black screen of 45 mm length; the spatial organization is illustrated in Figure 2. Below each white flicker light source we placed a green color standard signaling LED to instruct the subject where to look during the BCI task. The distance of the LED stimulation panel to the subject was kept ~60 cm, resulting in a visual angle of ~3.8° for every light source.

#### BCI task

The BCI task consisted in subjects trying to follow a prefixed sequence of 16 steps by focusing their vision onto a specific flickering white light source out of the four possible ones at each step, as continuously indicated by the smaller green signaling LEDs below. This sequence was identical for all subjects. A brief beep sound confirmed the indicated flickering light source as correctly detected.

### Stimulation

We compared the BCI efficiency under three conditions of flicker frequency selection: (i) by the assisted closed-loop (*ACL*) protocol, (ii) by a standard protocol with stimulation frequencies *prefixed* at 27, 28, 29, and 30 Hz (because 1 Hz distances are commonly employed in SSVEP-BCIs e.g., Herrmann, 2001; Diez et al., 2011; Volosyak et al., 2011), and (iii) by a protocol which used a selection of *top* frequencies for each subject (see section “ACL Algorithm”). In order to compensate for possible presentation order effects, the order of (i), (ii), y (iii) was permutated over the subjects.

**Figure 3:**
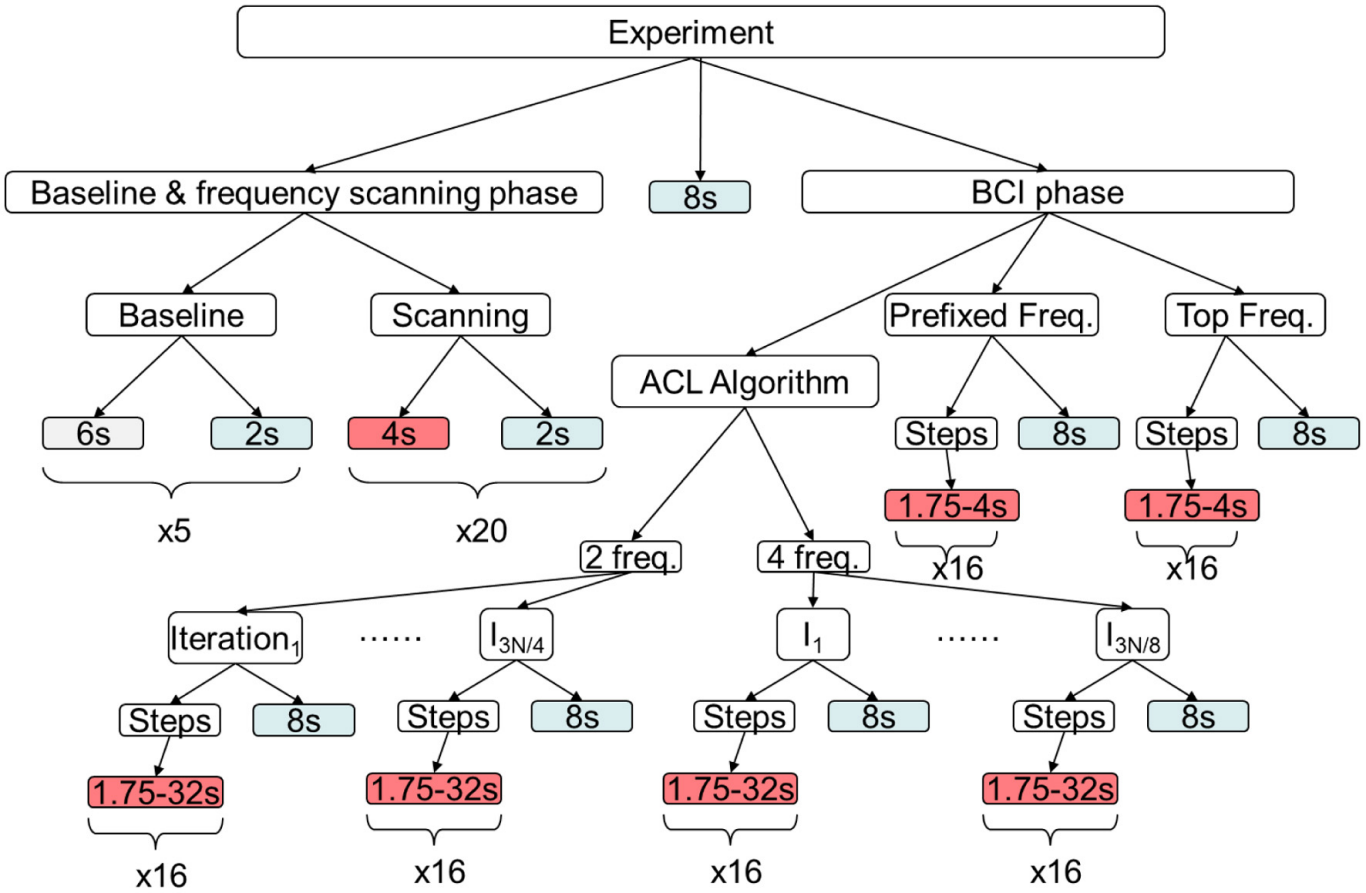
**Timeline of the experiment.** In the first phase individual EEG *baseline* activity is measured and in the following *frequency scanning phase* those frequencies electing largest SSVEP magnitudes are selected for each subject individually, while those below a predefined threshold are excluded (*Top Freq.*). Later, these values are used in the BCI phase. Under the *prefixed frequency* condition, always the same frequency set of 27, 28, 29, and 30 Hz is used for stimulation. *Red* boxes indicate stimulation, *blue* resting periods and *gray* baseline recording; in each box durations are reported.

Figure 3 shows the timeline of the experiment. The first phase of the experiment consisted in the measurement of the individual EEG *baseline* and the *frequency scanning phase* to select a set of flicker stimulation frequencies for each subject (the number of frequencies in this set is specific for each participant—see below). The second phase is the BCI phase with its three conditions (i), (ii), and (iii) mentioned above.

### Signal acquisition and preprocessing

**Figure 4:**
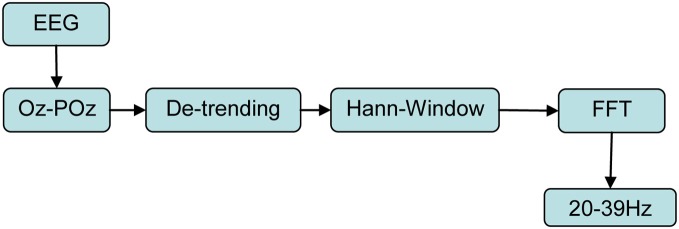
**Signal chain of acquisition and *online* preprocessing.** Input signals are the *time domain* EEG signals at electrodes Oz and POz sampled at 1024 Hz which finally result in normalized SSVEP spectral power densities *S*_*f*_ for each of the 20 stimulation frequencies *f* using as transformation to *frequency domain* the Fast Fourier Transform (FFT).

The signal acquisition and preprocessing steps are summarized in Figure 4. The EEG signal was recorded at 1024 Hz with eight sintered Ag/AgCl electrodes mounted into a “*Aegis Array*” stretch lycra cap (*Sands Research Inc.*, Texas/USA) using a “*BRAINBOX® EEG-1166*” 64 channel EEG amplifier (*Braintronics B.V*, Almere/Netherlands) with in-house software written in *C*. Vertical and horizontal EOG was recorded bipolarly by an in-house battery driven analog amplifier following a circuitry of Usakli and Gurkan (2010) with sintered Ag/AgCl electrodes fixed by adhesive rings above/below the left eye vs. at left/right *epicanthus* connected to a data acquisition board (*NI-PCI-6251, National Instruments*) at 1024 Hz. The eight standard 10–20 positions were FPz, F3, Fz, F4, Cz, Pz, POz, and Oz (Jasper, 1958). For *online* SSVEP detection as BCI input only POz and Oz were used, while for later *offline* studies the signals from *all* eight mentioned electrodes were analyzed. The EEG reference electrode was placed at nose tip, EOG ground electrode at *glabella* and impedances were kept <10 kΩ.

**Figure 5:**
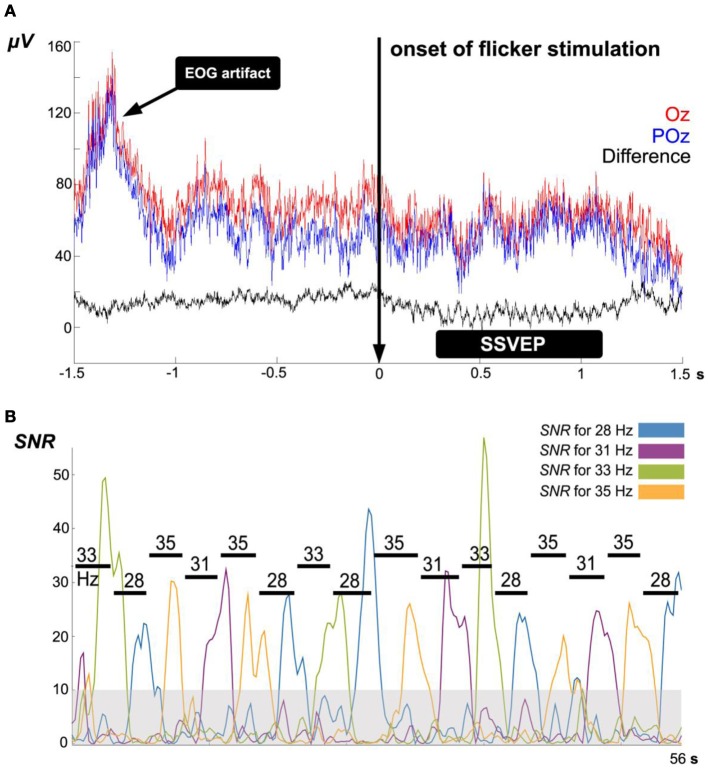
**(A)** Example of EEG *time domain* signals during 3 s before and after 21 Hz flicker stimulation at electrodes Oz (red) and POz (blue). Using their difference signal (black) as BCI input, in the sense of a *bipolar montage*, remarkably reduces common DC offsets, EOG/EMG artifacts and EEG contributions other than due to the visual cortex: the difference signal offers a simple *spatial high-pass filter.* **(B)** Example of signal-to-noise ratios *S*_*f*_ during a single iteration of the algorithm ACL using four different flicker frequencies. The gray shadowed area represents the *noise floor* with dimensionless value 10; this level was defined as SSVEP detection threshold for all subjects. Horizontal lines indicate the detection duration of each target frequency at each *step.*

To improve SSVEP detection, we used the online computed difference signal between Oz and POz as bipolar montage as the only input signal to our BCI system. This reduces both EOG/EMG artifacts and EEG activity not related to the visual cortex because this montage implements a simple and computationally inexpensive *spatial high pass filter* (see Figure 5). Thus, the *SNR* for the SSVEP detection is increased as compared to unipolar montages (Diez et al., 2010). In a time window of 2 s, this difference signal was then linearly detrended, treated by a *Hann*-window and then converted into *frequency domain* by Fast Fourier Transform (FFT) with a window length of 2048 sample points. The chosen *Hann-window function* has a quite narrow main lobe, which determines a good frequency resolution, and reasonable side lobe suppression (Harris, 1978). Those FFT coefficients meeting the exact flicker frequencies were used, one single coefficient for each flicker frequency. Thus, 20 real numbers were obtained and squared to represent the *power spectral densities* (PSDs) in the flicker range 20–39 Hz (see Figure 4). This procedure was developed following Diez et al. (2011). The described analysis was continuously repeated as *sliding windows* with a displacement of 250 ms, resulting in 87.5% overlapping. With all four LEDs emitting steady light, magnitudes of baseline EEG activities *B*_*f*_ were measured over 30 s at each future flicker stimulation frequency, determined as *M*_PSD_ by the described procedure (5 sets of 6s with 2 s resting periods in between, see Figure 3 *Baseline*). Subjects were instructed to use only the resting periods in-between for eye blinks/relaxation and otherwise maintain their eyes quietly open, trying to avoid jaw and tongue movements to reduce EOG/EMG artifacts.

For the *frequency scanning phase* of the experiment an identical measurement procedure was used, but with time windows for flicker stimulation of 4 s in each frequency *f* of the 20–39 Hz range resulting in magnitudes of SSVEPs as response, *R*_*f*_. Each stimulation epoch is followed by a 2 s resting period. In the *BCI phase* of the experiment, the same procedure is used for the selected stimulation frequencies in a single measurement window of 2 s.

SSVEP PSD magnitudes were normalized to EEG baseline activity in a given frequency *f* as dimensionless *signal-to-noise* ratios:

(1) $$S_{f}=R_{f}/B_{f}$$

In order to minimize fatigue, we tried to keep the baseline and frequency scanning phase as short as possible, 40 s in total for the baseline and 160 s for frequency scanning.

### ACL algorithm

#### Selection of the top frequencies for each subject

A closed-loop approach is used to select the set of the four *top* stimulation frequencies by compatibility for each subject and in the given experimental context. As a first step, the specified range is scanned which results in a-priori score for each of them. Stimulation frequencies are defined as valid if their *S*_*f*_ exceeds a prefixed threshold (set to 10) any time during the ongoing flicker stimulation. For *N* valid frequencies, the frequency corresponding to the largest *S*_*f*_ gets an initial score of *s*_1_(0) = *N*, the second to best *s*_2_(0) = *N* − 1, etc. The frequency corresponding to the lowest *S*_*f*_ gets a score of *s*_*N*_(0) = 1. Finally, the four best scores define the selection of the four *top* stimulation frequencies.

#### First closed-loop in the ACL-algorithm: iterative selection of the most compatible frequencies

The previous procedure provides initial scores for each frequency *s*_1_(0), *s*_2_(0),…, *s*_*N*_(0) which depend on subjects' intra- and interindividual *state* and *trait* dimensions and on the extrinsic conditions in which the BCI is used. The selection of the four stimulation frequencies is then further optimized in an iterative approach attending to their compatibility. Thus, as the next step, we calculate the following compatibility measure between all possible pairs of frequencies *x* and *y* taking into account a measure of their distance and their scores:

(2) $$c_{xy}(t)=\alpha \cdot \left(s_{x}(t)+s_{y}(t)\right)+\beta \cdot d_{xy}$$

Here *t* represents the iteration number. We assigned the following weights to the distance and the scores: α = 1.5 and β = 1, respectively, where *d*_*xy*_ is a measure of the distance between the frequencies which we define below. The values for α and β were set empirically based on several trials. Because four frequencies are used simultaneously in our specific BCI implementation, the most compatible four frequencies have to be selected out of *N* valid frequencies, determined by the protocol described above: the first step is to identify pairs of frequencies with optimal compatibility (“2 freq.” search in the ACL branch in Figure 2). This search consists of *3N/4* iterations (see below), each of them divided into 16 steps with a resting period at its end. The ACL departs from the scores calculated in the scanning procedure *s*_1_(0), *s*_2_(0),…, *s*_*N*_(0): they are modified in the successive iterations to search for the best compatibility.

In each iteration, the subject has to follow a sequence of flicker light sources by focusing upon them, as continuously indicated by the location of the green light. The flicker frequencies are chosen by selecting *max*_*xy*_(*c*_*xy*_) at the end of the iteration. To update the scores, we take into account both the success rate and the time as:

(3) $$s_{x}(t)=s_{x}(t-1)\cdot (\delta \cdot \text{SR }-\;\gamma \cdot T)$$

where SR is the success rate (correct SSVEP detections over 16, the number of possible detections) and δ and γ are parameters of the ACL algorithm which were set to δ = 1.2 and γ = 0.02. *T* is the duration of the detection in seconds. The values for δ and γ were chosen based upon the range of SR and *T and several simulations.*

In this first part of the algorithm, the distance between two specific frequencies *f*_*x*_ and *f*_*y*_ for Equation (2) is calculated as:

(4) $$d_{xy}=\left|f_{x}-f_{y}\right|$$

Each *c*_*xy*_(*t*) is updated by the new scores after each iteration. Once this procedure has run *p* = ⌊3N/4⌋ times, the highest *c*_*xy*_(*p*) is selected and a new set is created with the union of both frequencies. Now, the next highest *c*_*x′y′*_(*p*) disjoint from the previous set is chosen and a new set is constructed. This is repeated ⌊N/2⌋ times because this is the total number of possible disjoint pairs. It is ensured that each set is disjoint from all others. *p* = ⌊3N/4⌋ is chosen to test ⌊3N/2⌋ frequencies, so that the best frequencies are tested more than once. It is important to note that the duration of the frequency tests has to be restricted.

Afterwards, the second part of the algorithm is performed, the selection of four frequencies. The same procedure as in the first part is employed, but instead of single frequencies, sets of two frequencies are used. The values of *s*_*x′*_(*p* + 1) of each set are adjusted according to the values *c*_*xy*_(*p*), where *x*′ = *x* ∪ *y*. In this way, the set with the highest value gets *s*_1′_(*p* + 1) = ⌊N/2⌋, the second best *s*_2′_(*p* + 1) = ⌊N/2⌋ − 1 and so on. The last one gets *s*_⌊N/2⌋′_ (*p* + 1) = 1. From this point of the algorithm on, these sets are indivisible.

Using the same procedure performed with two frequencies, the process is repeated with four of them. The compatibility and the score actualization rules are still the same. The only difference is the distance measure for Equation (2) calculated as:

(5) $$d_{xy}=\frac{\sum_{i=1}^{2k}\sum_{j=1}^{2k}\left|f_{i}-f_{j}\right|}{2k\cdot (2k-1)}$$

where *k* is the number of frequencies of each set (in this case 2), and *f*_*i*_ and *f*_*j*_ are the individual frequencies taken from the union of the sets *x* and *y*. Note that here *x* and *y* refer to sets of two frequencies while in Equation (4) *x* and *y* referred to individual frequencies. This distance expresses the arithmetic mean of all possible pairs in the set resulting from the union of the initial sets *x* and *y*. Note that for *k* = 1, this distance measure is exactly the same distance (Equation 4) as used in the first part of the algorithm. In this second part ⌊3N/8⌋ iterations are performed, which is *N*/2 (the number of disjoint sets) times 3/4 (see above).

#### Second closed-loop in the ACL-algorithm: online auditory feedback of SSVEP magnitudes

In order to offer additional dynamic information to the subject related to his/her brain activity beyond the SSVEP detection confirmation cue, we provide a continuous online auditory feedback during the trials which represents the distance between the actual state and the pre-defined goal. The feedback signal consists of a 20 possible sinusoids with a range between 100 and 575 Hz which are updated every 0.25 s. The represented distance measure is defined as the difference between the EEG-SSVEP *signal to noise ratio* for the target frequency (S^target^_*f*_) and the threshold. Once S^target^_*f*_ has reached this threshold level, the auditory feedback is muted. Previously, subjects are instructed that their goal is to raise the pitch of the sinusoids as high as possible, and that after possible success their further goal would be trying to keep the sounds muted for 1.75 s; after this silence, the program automatically proceeds to the trial's next step. This kind of continuous auditory feedback aims to help subjects to learn to gain control *in their particular way* over SSVEP magnitudes by attracting their attentional resources to these voluntary attempts to increase self-regulation of their resonating brain states.

Concluding, there are two assisted closed loops in our system: the first one operates over the stimulation frequency set with the aim to directly improve the ITRs of each subject. This closed-loop informs the system about subject and environment specificities. The second one informs the subject about his/her brain activity in relation to the use of the interface and helps him/her to do so faster and more accurately. This closed loop works several times for each step of a trial.

### SSVEP detection

In order to reduce the experiment's complexity in terms of a reductionistic paradigm, we choose a simple SSVEP detection strategy in our study. During the *top* and *prefixed* frequency stimulation, the S^target^_*f*_ value is calculated every 0.25 s. If this value exceeds the threshold for 1.75 consecutive seconds, then this SSVEP is defined as “detected.” The threshold value was set to 10 which reflects the observed noise flow (see Figure 5). To avoid longer waiting periods when the subject is unable to exceed the threshold, a time limit of 4 s is used, after which that step is considered as fault.

During the ACL, to favor SSVEP detection in case that the subject exceeds the threshold and more time than the 1.75 s is needed to be classified as “detected,” there is a small modification in this protocol to allow adaptive time extensions. When S^target^_*f*_ exceeds the threshold in a given 0.25 s time step, the time limit is increased for another 0.25 s.

### Efficiency measures

After each iteration of the algorithm, both the success rate and time needed are saved. For the *prefixed* and *top* frequencies, *standard Information Transfer Rate* (ITR) is calculated:

(6) $$\begin{array}{l} \text{ITR}(SR,\;t)=({\text{log}}_{2}(N)+SR\cdot {\text{log}}_{2}(SR)+(1-SR)\cdot \\ \;{\text{log}}_{2}((1-SR)/(N-1)))\cdot Norm/t \end{array}$$

where *N* is the number of targets (*N* = 4 in our case). The value *SR* represents the success rate and *t* is the time taken in minutes. *Norm* is a normalization value set to 960 (60 s times 16 steps in each iteration). Note that if *SR* ≤ 1/*N*, then ITR(*SR, t*) = 0.

In contrast to the conditions *prefixed* and *top*, ITR is measured *several* times during the ACL. Thus, for further a-posteriori analyses these ITR distributions have to be represented by descriptive statistics: for condition *ACL* therefore *M* and *Mdn* of success rates and needed times are used to calculate ITR_Mean_ and ITR_Median_, completed by maximum ITR (ITR_Max_).

### Convergence measure

For a-posteriori analyses, a *convergence measure* for the algorithm in terms of the stimulus frequency exploration was defined: the duration of the 2 freq. search of the algorithm is divided into two parts. For each part, the numbers of explored frequencies are determined and divided by the maximal number of possible frequencies which could be explored (twice the number of iterations). The decrease comparing this measure in the second part vs. in the first part is a sign for how much the frequency exploration is converging. As can be seen in Table 1, the number of iterations varies over the subjects. The convergence measure is not reported for the first part because in our sample all subjects had the same maximal value 1, i.e., all possible frequencies were explored. We will use this measure to discuss how the ACL algorithm seems to adapt to subjects' interindividual differences.

**Table 1:** **Data of the *N* = 18 subjects under the three experimental conditions**.

**No. of subject**		**SR**	**SR**	**SR**	**SR**	**SR**	**ITR**	**ITR**	**ITR**	**ITR**	**ITR**	**Age**	***SNR* SSVEPs in scanning phase**	**Convergence measure**	
		**Pre**	**Top**	**Mean**	**Mdn**	**Max**	**Pre**	**Top**	**Mean**	**Mdn**	**Max**		
				**ACL**	**ACL**	**ACL**			**ACL**	**ACL**	**ACL**			
														***N* trials**	**2nd half**
1		0.31	0.88	0.77	0.78	0.88	**0.24**	**21.19**	**15.01**	**15.34**	**21.57**	23	15.20	13	0.5
2		0.56	0.63	0.57	0.5	0.75	**5.65**	**7.34**	**5.03**	**3.06**	**11.7**	23	15.89	11	0.4
3		0.75	0.38	0.80	0.88	0.94	**11.88**	**0.82**	**15.25**	**19.48**	**26.33**	27	14.44	14	0.57
4		0.81	0.94	0.95	0.97	1	**17.89**	**27.29**	**26.62**	**29.21**	**34.9**	33	31.38	15	0.71
5		0.75	0.56	0.68	0.59	0.69	**16.9**	**7.09**	**12.08**	**8.42**	**12.98**	24	8.02	15	0.36
6		0.06	0.25	0.35	0.25	0.63	**0**	**0**	**0.56**	**0**	**6.98**	25	8.08	9	0.5
7		0.81	0.69	0.85	0.81	1	**19.32**	**11.68**	**21.18**	**18.58**	**36.92**	59	23.76	12	0.92
8		0	0.44	0.59	0.59	0.75	**0**	**1.88**	**5.12**	**5.12**	**10.56**	18	8.03	5	0.5
9		0.63	0.44	0.69	0.69	0.75	**6.76**	**1.79**	**9.17**	**9.17**	**12.47**	52	8.68	4	1
10		0.75	0.75	0.83	0.84	0.94	**16.53**	**16.18**	**21.16**	**22.31**	**30.02**	23	59.20	14	1
11		0.56	0.81	0.88	0.88	1	**5.35**	**18.23**	**21.84**	**22.16**	**31.47**	50	37.70	14	0.64
12		0.19	0.25	0.56	0.69	0.69	**0**	**0**	**4.67**	**9.42**	**9.73**	24	6.35	6	0.33
13		0.69	0.81	0.81	0.81	0.94	**10.07**	**19.32**	**18.06**	**18.06**	**28.32**	34	8.43	8	0.87
14		0.69	0.75	0.66	0.69	0.75	**12.7**	**16.53**	**11.13**	**12.7**	**16.18**	27	48.58	14	0.93
15		0.31	0.31	0.58	0.56	0.69	**0.21**	**0.22**	**5.4**	**4.96**	**9.57**	45	12.01	14	0.43
16		0.18	0.5	0.63	0.56	0.94	**0**	**3.43**	**6.34**	**4.42**	**21.14**	20	16.35	9	0.38
17		0	0	0.34	0.34	0.38	**0**	**0**	**0.42**	**0.42**	**0.76**	22	14.15	11	0.6
18		0.5	0.69	0.76	0.75	0.81	**3.43**	**12.98**	**15.43**	**14.63**	**18.58**	32	30.03	13	0.67
*Shapiro–Wilk's*	*W*	0.883	0.961	0.947	0.952	0.896	0.850	0.886	0.950	0.960	0.956	0.832	0.819	0.876	0.909
	*p*	0.030	0.631	0.385	0.460	0.049	0.008	0.034	0.420	0.594	0.520	0.005	0.003	0.022	0.082
***Mdn***		**0.56**	**0.60**	**0.69**	**0.69**	**0.78**	**5.5**	**7.22**	**11.61**	**11.06**	**17.38**	**26.00**	**14.82**	**12.50**	**0.59**
*Percentile 25*		0.19	0.36	0.58	0.56	0.69	0.00	0.67	5.10	4.83	10.35	23.00	8.34	8.75	0.42
*Percentile 75*		0.75	0.77	0.82	0.82	0.94	13.66	16.96	18.84	18.81	28.75	36.75	30.37	14.00	0.88

Note: Information transfer rates (ITRs) in bits/min as measures of individual BCI performances under the different experimental conditions and all Mdn values are highlighted in bold for further analyses.

N trials refers to the number of iterations in the first part of ACL (using two flicker LEDs).

Convergence measure first half is not reported in the table because all subjects had the same value 1.

SNR SSVEPs in Scanning phase are means over all used 20 flicker frequencies.

### Study design

A three conditions (*ACL, top, prefixed*) balanced *within*-subjects design with three times full permutation of presentation order (ABC, ACB, BAC, BCA, CAB, CBA) and with random assignment of subjects, resulting in *N* = 18 was employed.

### Baseline resting state EEG measures as possible interindividual correlates of ITR performances

Aiming to investigate possible correlations between baseline resting state EEG measures and the variables of the experiment, the 30 s baseline EEG (see Figure 3) at all eight electrodes reported above were manually cleaned from artifacts with the result of *M* = 20.02 s, *SD* = 5.54 artifact free epochs. Under *MATLAB 7.11.0.584 win64*, EEG signals were preprocessed in a first step by linear detrending followed by a 8th order *Butterworth* 1.5–70 Hz band pass filter and finally by a 8th order *Butterworth* 45–55 Hz notch filter against 50 Hz power line electromagnetic interferences. Then, preprocessed EEG signals were converted into *frequency domain* by a sliding windows FFT transform of 2 s window length (2048 sample points) with 3.906 ms displacement (4 sample points, which correspond to a 256 Hz sample frequency in the resulting frequency domain signals), after linear detrending and treatment by a *Hann*-window function. Obtained FFT coefficients were squared to obtain the power spectrum and then normalized by dividing by 2048 sample points. In order to obtain *absolute PSD*s for the defined EEG frequencies bands of interest, corresponding coefficients were summed: *thetaLow* (3.5–6.5 Hz), *thetaHigh* (6.5–7.5 Hz); *alphaLow* (7.5–9 Hz), *alphaHigh* (9–12.5 Hz); *betaLow* (12.5–18 Hz), *betaMid* (18–24 Hz), *betaHigh* (18–30 Hz); *totalSpectrum* (0.5–70 Hz). In a first step, those absolute frequency domain *PSDs* signals were normalized dividing every sample point by the corresponding one of *totalSpectrum* which resulted in dimensionless ratios. These ratios indicate for every 256 time points per second the *relative* energy contribution of the frequency band of interest to the EEG total energy at this particular moment. In a last step, in order to represent EEG baseline resting state activities in the analyzed artifact free epochs by one single value for every frequency band, means of these normalized signals were computed over all corresponding time points. Thus, finally we obtained the desired baseline resting state EEG measures as *relative mean PSDs* for further correlational analyses, single values for every frequency band over all subjects.

Another measure of interindividual EEG variability is the resting state *individual alpha frequency* (IAF), because it has been found to be remarkably stable *within* subjects, but relatively variable *between* subjects (Kondacs and Szabó, 1999). In order to determine IAF in our experiment, coefficients of PSDs corresponding to the frequency band 8–13 Hz at *Oz* were normalized by *totalSpectrum* PSDs and averaged over all sliding windows in the artifact free baseline resting state epochs. In this averaged and normalized power spectrum the *alpha* frequency with the highest PSD was manually measured and defined as IAF (*peak frequency method*).

### Statistical analyses

All statistical analyses were computed using SPSS 17.0 and STATISTICA 6.0. Previously, *Shapiro–Wilk* tests were calculated to check each of the three conditions for normal distribution in the underlying populations. If one or more conditions showed significant departures from normality, *non-parametric* tests were preferred for further analyses: a *Friedman* test was performed as an *omnibus* test to investigate whether the *central tendencies* of one or more conditions differed significantly from the rest. In case of such a significant result, *post hoc pairwise comparisons* were performed in order to find out what conditions exactly differed significantly from each other, based upon comparison of *mean rank differences* using as significance criteria the *critical rank differences* proposed by the more progressive approach of Conover (1980) vs. the more conservative of Schaich and Hamerle (1984).

In order to quantify the *effect sizes* of those *post hoc* pairwise comparisons which resulted in significant differences, we used the probability of superiority of dependent scores, *PS*_*dep*_, recommended by Grissom and Kim (2012) and developed in Grissom (1994). It expresses the probability that in a randomly sampled matched pair the value from the condition containing the higher scores is indeed larger than that from the one containing lower scores. *PS*_*dep*_ is calculated by dividing the number of *positive* differences between the condition *containing the higher scores* minus the condition *containing the lower scores* by the total number of matched pairs. For classifying *PS*_*dep*_ into small, middle and large effect sizes based upon the standards of Cohen (1988), the *cut-off* values reported by Grissom (1994) are used: *small* 0.56, *medium* 0.64, and *large* 0.71. The same author offers a table to directly convert *PS* into equivalent *Cohen's* Δ. Thus, as effect size measures both *PS*_*dep*_ and *Cohen's* Δ are reported with standards *small* Δ = 0.20, *medium* Δ = 0.50 and *large* Δ = 0.80 (Cohen, 1988).

In order to check whether significant differences over all six possible permutations of the presentation order might be found, a *mixed-design repeated measures ANOVA* was computed with *stimulation condition* as repeated *within*-subjects factor with three levels (i) ACL algorithm represented as ITR_*Median*_, (ii) prefixed and (iii) top and *presentation order* as *between*-subjects factor with the six possible permutations as levels (ABC, ACB, BAC etc.). Previously, *Levene's* tests were performed in order to check for homogeneities of error variance. Moreover, the assumption of *sphericity* of the covariance matrix was verified previously by a *Mauchly's sphericity test* in order to assure that the *F* ratios match an *F* distribution. If there was a significant departure from sphericity, *Greenhouse-Geisser* estimates were used to correct degrees of freedom which results in fractions instead of usual integers. Although data may not follow a normal distribution, *ANOVA* has been demonstrated to be relatively robust against moderate deviations from normality (see e.g., Khan and Rayner, 2003). Univariate analyses were used to examine whether there is a significant *between*-subjects main effect of *presentation order* and further if there is a significant *interaction* effect between *presentation order × stimulation condition*. Analyses were repeated representing condition (i) ACL algorithm also as ITR_Mean_ vs. ITR_Max_.

For the investigation of linear correlational relationships, *Spearman's rank order correlation coefficient Rho* was additionally used apart from the common *Pearson product-moment correlation coefficient r* due to its relative robustness firstly against outliers, but also against other than linear, but still monotonic relationships and against departures from normality or homoscedasticity. Whenever relevant influence of outliers was suspected, *Spearman's rank correlation coefficient Rho* was preferred.

A-priori *statistical test power* analyses with the program G^*^Power 3 (Faul et al., 2007) show that *Pearson* correlation significance tests in the employed sample size of *N* = 18 and with standard significance level α = 0.05 have test powers (1 − β) ≥ 0.80 as recommend by Cohen (1988), when they have effect sizes in the underlying population ρ ≥ 0.60, as compared to *H*_0_: ρ = 0.00. For ρ = 0.50 test power is (1−β) ≥ 0.60, for ρ = 0.40 (1−β) = 0.40 and for ρ = 0.30 (1−β) ≈ 0.20. Thus, although the employed sample size *N* = 18 is relatively small, hypothesis testing of *Pearson* correlations with full recommended strictness is definitely possible at the level of assumed large effect sizes.

## Results

Table 1 reports the data for all *N* = 18 subjects under the three experimental conditions, representing (i) *ACL algorithm* as ITR_Mean_, ITR_Median_ and ITR_Max_. Inferential statistical hypotheses testing that (i) outperformed the other two flicker stimulation conditions is reported below.

**Figure 6:**
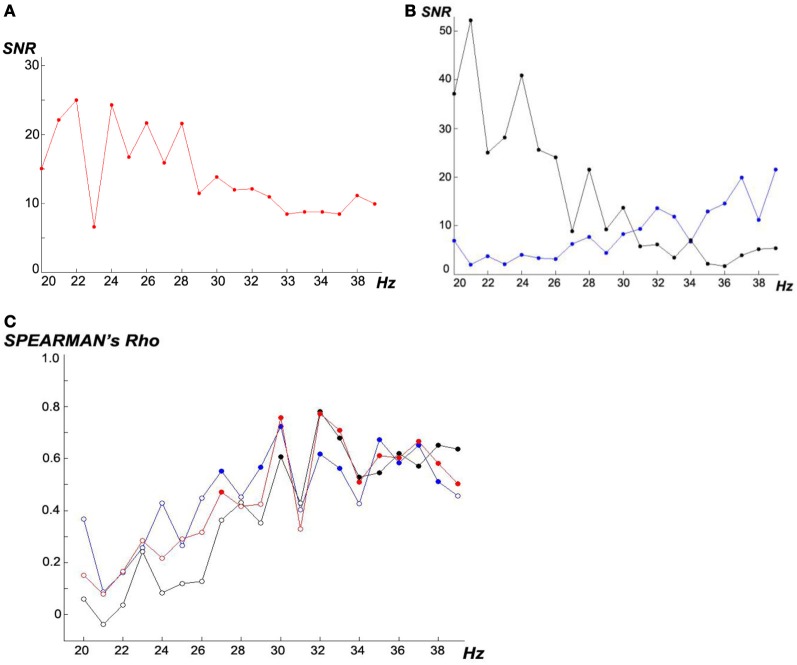
**(A** and **B)** SSVEP-*SNR* frequency-response curves. **(A)** *Mdn*s over all *N* = 18 subjects, **(B)** example of two subjects with opposed frequency-response curves (black # subject 16, blue #9). **(C)** Frequency-dependent interindividual association between SSVEP-*SNR* magnitudes and ITR performances under the three experimental conditions, computed as *Spearman's* rank order correlations: (i) *ACL algorithm* (red), (ii) *top* (blue) and (iii) *prefixed* (black), filled circles represent significant *p* < 0.05.

Figure 6 shows the SSVEP frequency-response curves in our experiments. For all subjects, the 20 flicker frequencies in the scanning phase were presented in the same order: 23, 37, 30, 31, 36, 22, 29, 33, 39, 24, 35, 21, 25, 27, 32, 34, 28, 20, 26, and 38 Hz. Sequential randomness of this order is confirmed with *Z* = −0.230 and *p*_exact_ = 0.828 (*Wald–Wolfowitz* runs test after *Mdn* split dichotomization). Our findings that in the 20–39 Hz range, lower flicker frequencies *over all subjects* (Figure 6A) evoke higher SSVEP magnitudes are in line with other studies which reported a global maximum SSVEP amplitude around 10 Hz with additional local maxima around 20, 40, and 80 Hz (Regan, 1989; Herrmann, 2001; Bayram et al., 2011). In our sample, we found that SSVEP frequency-response curves differed remarkably *between subjects* (Figure 6B) probably due to trait and state variabilities which justifies that they are determined in our experiment in the scanning phase for every subject *individually.*

Analyzing Figure 6C, higher frequencies ≥30 Hz lead to higher correlations; no relevant differences can be seen comparing the three experimental conditions. Interestingly, following e.g., Zschocke and Hansen (2012), 30 Hz is the upper boundary of *beta* activity observable in scalp EEGs by conventional amplifiers.

### Significant and large improvement of SSVEP-BCI efficiency by the novel ACL algorithm

Analyzing the differences in the *central tendencies* between the three experimental conditions (i) *ACL algorithm* (ii) *prefixed* (iii) *top* we represented condition (i) based upon three different descriptive statistics, (a) ITR_Mean_, (b) ITR_Median_, (d) ITR_Max_ (see section “Materials and Methods” and Table 1). Applying non-parametric inferential statistics we found a very significant and very large superiority of condition (i) *ACL algorithm* over the other two (ii) and (iii) which is independent of its three types of representation (a), (b), and (c), while there is no significant difference between (ii) and (iii). The used statistical methods and measures for the following results are found in section “Statistical Analyses.”

(a) A *Friedman* omnibus test comparing the ITRs between the three experimental conditions (i) *ACL algorithm* **represented as ITR_Mean_**, (ii) *prefixed* and (iii) *top* shows a significant overall difference with χ^2^(2) = 10.116, *p* = 0.006.
  *Post-hoc pairwise comparisons* based upon *critical mean rank differences* 0.82 (Schaich and Hamerle, 1984) vs. 0.58 (Conover, 1980) indicate that ITRs are significantly higher in (i) *ACL algorithm* as compared to (ii) *prefixed* (*mean rank difference* = 1.03, very large effect size *PS*_*dep*_ = 0.83, Δ = 1.37) and also as compared to (iii) *top* (*mean rank difference* = 0.64, large effect size *PS*_*dep*_ = 0.72, Δ = 0.83). Comparison of (ii) *prefixed* with (iii) *top* results in a non-significant difference (*mean rank difference* = 0.39).
(b) A *Friedman* omnibus test comparing the ITRs between the three experimental conditions (i) *ACL algorithm* **represented as ITR_Median_**, (ii) *prefixed* and (iii) *top* shows a significant overall difference with χ^2^(2) = 9.262, *p* = 0.01.
  *Post-hoc pairwise comparisons* based upon *critical mean rank differences* 0.82 (Schaich and Hamerle, 1984) vs. 0.57 (Conover, 1980) indicate that ITRs are significantly higher in (i) *ACL algorithm* as compared to (ii) *prefixed* (*mean rank difference* = 0.94, very large effect size *PS*_*dep*_ = 0.81, Δ = 1.25) and also as compared to (iii) *top* (*mean rank difference* = 0.64, very large effect size *PS*_*dep*_ = 0.76, Δ = 1.21) applying the less conservative criterion of (Conover, 1980). Comparison of (ii) *prefixed* with (iii) *top* results in a non-significant difference (*mean rank difference* = 0.31).
(c) A *Friedman* omnibus test comparing the ITRs between the three experimental conditions (i) *ACL algorithm* **represented as ITR_Max_**, (ii) *prefixed* and (iii) *top* shows a significant overall difference with χ^2^(2) = 22.986, *p* = 0.00001.
  *Post-hoc pairwise comparisons* based upon *critical mean rank differences* 0.82 (Schaich and Hamerle, 1984) vs. 0.41 (Conover, 1980) indicate that ITRs are significantly higher in (i) *ACL algorithm* as compared to (ii) *prefixed* (*mean rank difference* = 1.47, extremely large effect size *PS*_*dep*_ = 0.94, Δ = 2.25) and also as compared to (iii) *top* (*mean rank difference* = 1.19, extremely large effect size *PS*_*dep*_ = 0.94, Δ = 2.25). Comparison of (ii) *prefixed* with (iii) *top* results in a non-significant difference (*mean rank difference* = 0.28).

### The ACL algorithm seems to adapt to subjects' interindividual differences

*N*_*Trials*_ in condition (i) *ACL algorithm* using two flicker LEDs (see Table 1) is deterministically given by 3/4 of the total number of the SSVEP-*SNR* responses under the 20 flicker frequencies in the scanning phase of the experiment which had exceeded the defined threshold value of 10 (*suitable* frequencies), see *ACL Algorithm* of section “Materials and Methods.” Thus, in order to make the investigation of possible interindividual associations between the SSVEP-*SNR* magnitudes with the convergence measure second half (see section “Materials and Methods”) relatively independent from *N*_*Trials*_, all subjects with *N*_*Trials*_ < 25th percentile (8.75 ≈ 9) were excluded, # subject 6, 8, 9, 12, 13, and 16. The resulting rest of *N* = 12 subjects showed a relatively small variability with range of *N*_*Trials*_ between 11 and 15. The measure SSVEP-*SNR* mean magnitudes in the scanning phase of the experiment (a) over all flicker frequencies from 20 to 39 Hz was split into two measures, one for (b) *lower* frequencies from 20 to 29 Hz and the other for (c) *higher* frequencies from 30 to 39 Hz. In this subsample, convergence measure second half shows large and highly significant correlations with (a) of *r* = 0.839, *p* = 0.001, with (b) of *r* = 0.843, *p* = 0.001 and with (c) of *r* = 0.763, *p* = 0.004. Checking these relationships against the remaining variability of *N*_*Trials*_ and age as controlled third variables in *partial correlation* analyses, indeed no changes are observed; those found relationships can be considered as linearly independent from *N*_*Trials*_ and age. Hence, these findings show that the convergence of the *ACL* algorithm highly depends on the subjects' *trait* ability to generate higher SSVEP-*SNR* magnitudes, with no relevant differences observed between *lower* vs. *higher* flicker frequencies: focusing on a subsample with a more or less constant number of *suitable* frequencies, the ACL algorithm explored the more distinct frequencies in those subjects who displayed the *larger* SSVEP-*SNR* magnitudes in the scanning phase of the experiment.

In conclusion, these findings imply that the ACL algorithm shows a distinct exploration behavior for different subjects and thus indeed is able to adapt to subjects' interindividual differences. Whether this adaptation is the *cause* for the ACL algorithm's outperformance of (ii) *top* and (iii) *prefixed* cannot be examined in depth with the employed experimental design and has to be investigated in further studies.

### Baseline resting state EEG measures as correlates of interindividual differences

Searching for significant and relevant associations between interindiviudal variabilities of ITR performances under the three experimental conditions vs. of baseline resting state EEG relative mean PSDs in all computed frequency bands at all eight used electrodes, effects were only found in *thetaHigh* (6.5–7.5 Hz) and *betaMid* (18–24 Hz). In all the other bands nothing could be observed.

Whereas *Pearson* correlations showed no relationships between the resting state relative mean *thetaHigh* PSDs at *Oz* vs. ITRs in conditions (iii) *prefixed* (*r* = 0.034, *p* = 0.894) and (ii) *top* (*r* = 0.196, *p* = 0.436), a significant positive correlation with condition (i) *ACL algorithm* was found (*r* = 0.467, *p* = 0.048) representing the performance as ITR_Median_. Searching for similar relationships in the other seven used electrodes, no associations were observed; these effects exclusively occur at *Oz* in our sample. Following the effects size classifications of Cohen (1988), this correlation is to be considered as *moderate. Partial correlation* analyses confirmed that this correlation is linearly independent against age and all means of SSVEP-*SNRs* in the previous scanning phase of the experiment over (a) *all* 20 flicker frequencies, (b) also over the *lower* frequencies 20–29 Hz and (c) also over the *higher* frequencies 30–39 Hz.

At least in the examined sample, interindividual variability in relative mean *thetaHigh PSD* at *Oz* seems to differentiate between *ACL algorithm* and the other two conditions: the larger the observed relative mean PSDs among subjects in the baseline resting state are, the better will be their later SSVEP-BCI performance exclusively under the use of *ACL algorithm.*

**Figure 7:**
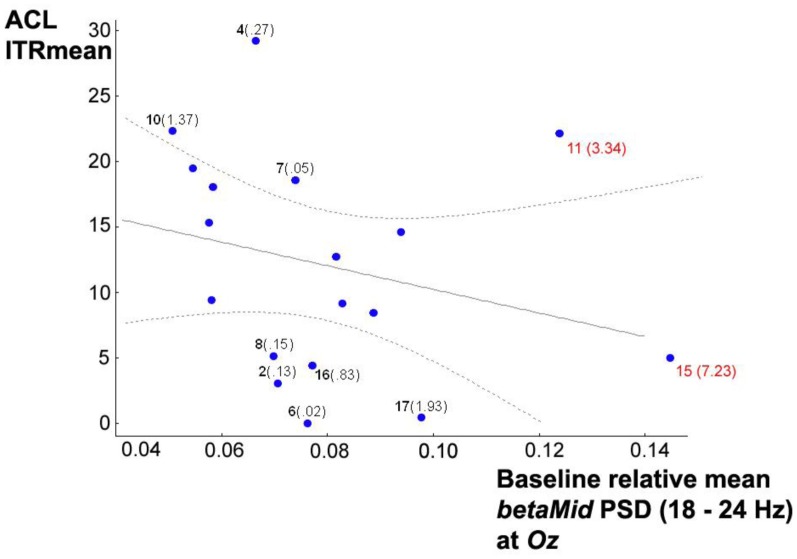
**Scatterplot of baseline resting state relative mean *betaMid* PSDs at *Oz* vs. ITR_Mean_ in condition (i) *ACL* algorithm, 95% confidence regression bands as dotted lines, subject numbers in bold, *Mahalanobis distances* in brackets calculated in a linear regression analysis with the ITR_Mean_ as criterion variables and relative mean *betaMid* PSDs as predictor variables.** Subject 15 and 11 (in red) might be considered as outliers (see text). Excluding them changes the *Pearson* correlation from *r* = −0.262, *p* = 0.294 to significant *r* = −0.510, *p* = 0.043.

At first sight, analyzing baseline resting state relative mean *betaMid* PSDs, an exclusive relationship with only the ITRs in condition (iii) *top* was found for *F3* (*r* = 0.484, *p* = 0.042), although its neighbor electrodes also showed relationships not very far away from significance, probably due to small sample size: *F4* with *r* = 0.425, *p* = 0.117 and *Fz* with *r* = 0.410, *p* = 0.091. All the other used electrodes showed no associations. After further graphic inspection of relevant scatterplots and *Box-Whisker-Plots*, a possible negative relationship between baseline resting state relative mean *betaMid* PSDs at *Oz* and ITR_Mean_ in condition (i) *ACL algorithm* was suspected, hidden by outliers. *Box-Whisker-Plots* suggested case 15 and 11 as outliers, so for further analysis *Mahalanobis distances* were computed in a linear regression analysis with the ITRs_Mean_ of condition (i) *ACL algorithm* as criterion variable and baseline resting state relative mean *betaMid* PSDs at *Oz* as predictor variable. The inspection of *Mahalanobis distances* and the scatterplot (see Figure 7) suggest that subject 15 and 11 might be considered as outliers. Excluding them changes the correlation from *r* = −0.262, *p* = 0.294 to significant *r* = −0.530, *p* = 0.042. *Partial correlation* analyses confirmed that this correlation is linearly independent against age and all means of SSVEP-*SNRs* in the previous scanning phase of the experiment (a), (b), and (c) mentioned above.

Interestingly, excluding case 15 and 11, baseline resting state relative mean PSDs *betaMid* vs. *thetaHigh* both at *Oz* show an almost significant correlation over the subjects with *r* = −0.482 and *p* = 0.059, probably due to the small sample size, which is stable against the third variables age and all SSVEP-*SNRs* in the previous scanning phase of the experiment (a), (b), and (c), mentioned above.

In conclusion, baseline resting state relative mean *betaMid* PSDs seem to predict ITR performances under (i) *ACL algorithm* vs. (iii) *top* in an opposed fashion depending on the electrodes: the *lower* baseline resting state relative mean *betaMid* PSDs are at *Oz*, the *higher* will be the ITRs under condition (i); and the *higher* baseline resting state relative mean *betaMid* PSDs are at frontal electrodes (*F3, Fz, F4)* the *higher* will be the ITRs under condition (iii). In addition to these findings in *betaMid*, the higher the baseline resting state relative mean *thetaHigh* PSDs at *Oz* are, the higher will be the ITRs exclusively under condition (i).

**Figure 8:**
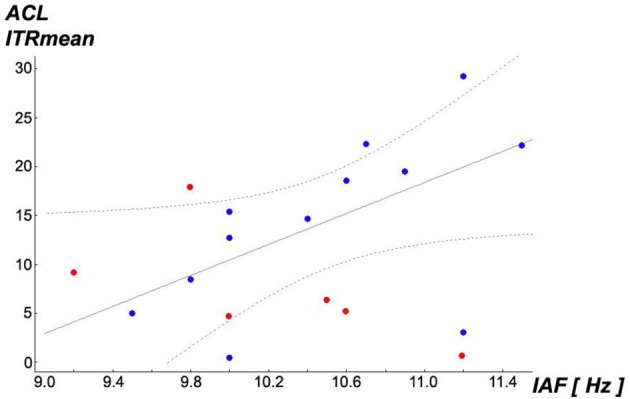
**Scatterplot of individual alpha frequency (IAF) vs. ITR_Mean_ under condition (i) *ACL algorithm* (best-fit regression line for *N* = 12 as continuous line, 95% confidence regression bands as dotted lines).** A significant *Pearson* correlation with *r* = 0.577, *p* = 0.0496 was found in the remaining subsample of *N* = 12 (blue points), removing subjects with *N*_*Trials*_ < 25th percentile (8.75 ≈ 9) (red points), while over the entire sample of *N* = 18 the correlation is hidden with *r* = 0.282, *p* = 0.257 (all points). This relationship seems to exist exclusively for condition (i) *ACL algorithm*: the higher subjects' IAF are in this subsample, the better will be their ITR_Mean_ performance exclusively under (i). *Partial correlation* analyses confirmed that this association is linearly independent against age.

Returning to the above described subsample of *N* = 12 obtained by exclusion of all subjects with *N*_*Trials*_ < 25th percentile (8.75 ≈ 9), an interesting observation was found: IAF shows differentiating relationships with ITR performances: a significant correlation of *r* = 0.577, *p* = 0.0496 was only found with ITRs under (i) *ACL algorithm* (see scatterplot Figure 8), but neither under (ii) *top* with *r* = 0.394, *p* = 0.205 nor under (iii) *prefixed r* = 0.283, *p* = 0.373. The higher subjects' IAF are in the subsample, the better will be their ITR performance exclusively under the ACL algorithm. *Partial correlation* analyses confirmed that this association is linearly independent against age. Repeating this analysis for the entire sample of *N* = 18 *no* significant correlations between individual alpha frequency (IAF) and ITR performances under the three experimental conditions become apparent (i) with *r* = 0.282, *p* = 0.257, (ii) *r* = 0.198, *p* = 0.432 and (iii) *r* = 0.243, *p* = 0.332. These findings imply that subjects with *low* ITRs in all three conditions might represent another population as compared to the rest. Further studies may try to replicate these findings and identify dimensions which discriminate between these possible two different populations. Moreover, these findings could be relevant for the understanding of the so-called *BCI illiteracy* phenomenon (Blankertz et al., 2010; Vidaurre and Blankertz, 2010; Volosyak et al., 2011), see section “Discussion.”

Inspired by the findings of Koch et al. (2008) who found correlations of IAF with both magnitudes of *visually evoked potential*s (VEPs) and also with cortical oxygenation measured by *near-infrared spectroscopy* (NIRS), *Spearman* rank order correlations were computed between IAF and means of SSVEP-*SNR* magnitudes in the scanning phase of the experiment (a) over *all* 20 used flicker frequencies 20–39 Hz, (b) over the *lower* frequencies 20–29 Hz and (c) over the *higher* frequencies 30–39 Hz in the described subsample of *N* = 12. Although not fully reaching significance level, probably due to the relatively small sample size, an interesting pattern was found: IAF vs. (a) with *rho* = 0.561, *p* = 0.058, IAF vs. (b) with *rho* = 0.183, *p* = 0.568 and IAF vs. (c) *rho* = 0.557, *p* = 0.060. Although not fully significant, probably due to the small sample size, interindividual differences in SSVEP-*SNR* magnitudes under the employed *higher* flicker frequencies seem to show a tendency of positive association to higher IAFs while this relationship might not exist for the stimulation with the *lower* frequencies (or if so, it may presumably be lower). These findings motivated the re-analysis of the found relationship in Figure 8 by *partial correlations* whether it would be linearly independent against SSVEP-*SNR* magnitudes in the scanning phase of the experiment (a), (b) and (c) as described above. While (a) and (b) showed no relevant influence on this relationship, controlling for (c) resulted in a reduction from former *Pearson r* = 0.577, *p* = 0.0496 to *r* = 0.396, *p* = 0.228. Hence, these findings imply that IAF and (c) the magnitude of SSVEP responses to only the employed *higher* flicker frequencies share remarkably amounts of common interindividual variability while explaining variability of ITR_Mean_ under the ACL algorithm.

### Effects of the permutation of presentation order

Investigating possible effects of the permutation of presentation order, a *mixed-design repeated measures ANOVA* was computed with *stimulation condition* as repeated *within*-subjects factor with three levels (i) ACL algorithm represented as ITR_Median_, (ii) prefixed and (iii) top and *presentation order* as *between*-subjects factor with the six possible permutations as levels (ABC, ACB, BAC etc.). *Levene's* tests showed homogeneities of error variances. There was no significant *between*-subjects main effect of *presentation order* with *F*_(5, 18)_ = 2.26, *p* = 0.115, η^2^_p_ = 0.485. Because *Mauchly's sphericity* test indicated a significant departure from the assumption of *sphericity* with χ^2^ (2) = 6.54, *p* = 0.038, *Greenhouse-Geisser* estimates were used to correct degrees of freedom (ε = 0.691). There was no significant *interaction* between *presentation order × stimulation condition* with *F*_(10, 18)_ = 0.67, *p* = 0.738, η^2^_p_ = 0.219. *ANOVA* analyses were repeated also for condition (i) ACL algorithm represented as ITR_Mean_ and ITR_Max_ which resulted in similar findings. In conclusion, neither significant main effects nor significant interactions could be found over all six possible permutations of presentation order. Hence, the found effects in the *central tendencies* reported above with regard to all ITR performances can be considered as independent from possible presentation order effects.

## Discussion

Although electrophysiology-based closed-loop interactions with biological nervous systems have been used since the 1940s, modern computers and online software control techniques allow a wide variety of novel activity dependent protocols in neuroscience research and related applications. Current BCI bring up a number of problems related to relatively long previous training times and still relatively low efficiencies (ITRs). This calls for novel techniques which can also address context and subject specificities, e.g., adaptive detection of SSVEPs (e.g., Krauledat et al., 2008).

In this paper we described an *assisted closed-loop protocol* which enhances BCI efficiency, as compared to classic BCI protocols, by providing both the subject and the system with online information which helps them to reach the BCI goal in their interaction. We used a reductionistic paradigm to constrain the inherent complexity of closed-loop exploration: four simultaneous frequencies, a basic SSVEP detection strategy and a relatively simple task to be accomplished by the user. More complex BCI systems might further benefit from the described approach. Our paradigm calls for many possible improvements, ranging from advanced SSVEP detection algorithms, stimuli which inform the user more effectively, up to a more adaptive online control of the interface itself by measuring and exploring additional dimensions (multimodality).

The literature on SSVEP-BCIs does not report general recommendations for the selection of the properties of the visual stimuli (Wu et al., 2008; Zhu et al., 2010), although it is known that the SSVEP magnitudes depend on extrinsic and intrinsic dimensions (Ding et al., 2006; Lopez-Gordo et al., 2011). Our study shows that a closed-loop subject-specific selection of the stimulation frequencies together with the closed-loop auditory feedback lead to increased BCI ITR performance which outperformed the employed control conditions.

Although *assisted closed-loop protocols* seem to enhance BCI efficiency, their use is limited by the additional time needed for the exploration process. In the protocol discussed in this paper, the average time to perform the experiment was around half an hour, flicker frequency selection took most of this time. Due to time restrictions, the parameter space can never be explored completely, so BCI efficiency improvement might remain suboptimal. Thus, there is some unknown *trade-off* between improvement and time needed, which should be explored in further studies. Furthermore, the question how replicable the found flicker frequencies are in the same subjects over multiple follow-up time points could be explored. Probably, observing this stability over time (e.g., *test-retest reliability*) may help to discover important trait vs. state dimensions related to variability of BCI performance. Another limitation due to the SSVEP physiology is that the time window for the auditory feedback is relatively short, so subjects have to establish control over the BCI goal in the range of a few seconds. This implies possible interactions with subjects' traits and states related to cognitive *processing speed* and dimensions of learning abilities.

ACL algorithms offer new possibilities as compared to traditional open-loop paradigms, but require additional decisions and new perspectives for their design and analysis, e.g., with regard to online measurement of actual states and performance, parameter search responding to the particular dynamic behavior of the system, properties of the feedback stimuli, *actuation laws*, etc. However, our findings imply that this additional effort can improve BCI efficiency and contribute to reveal dynamics of the nervous system which would remain hidden under traditional paradigms. Because our analyses showed that EEG resting state measures can predict assisted closed-loop SSVEP-BCI performance, our novel approach seems to flexibly adapt/interact with interindividual cerebral variabilities. Although found in the context of a *sensory motor rhythms* (SMRs) based BCI, other recent work also demonstrated that EEG resting state measures can be relevant predictors of BCI performance (Blankertz et al., 2010). In this emerging field, it could be fruitful to identify possible EEG resting state measures which can differentiate/predict between BCI performances based on biosignals originating from distinct physiological mechanisms: SSVEPs, P300**, SMRs, *slow cortical potentials* (SCPs)*, electrocorticogram* (ECoG), *magnetoencephalography* (MEG), NIRS *or blood-oxygen-level-dependent* (BOLD). Apart from these biosignals reflecting *brain* activity, *peripheral* psychophysiological measures have been investigated in the context of BCIs, especially as performance predictors, such as *parasympathic/vagal* parameters of resting state *heart rate variability* (HRV) (Kaufmann et al., 2011).

Our proposed approach of new adaptive-interactive paradigms might offer innovative ways how to address the problem of the so-called *BCI illiteracy*, i.e., the incapacity of some subjects to achieve control of BCIs (Blankertz et al., 2010; Vidaurre and Blankertz, 2010; Volosyak et al., 2011). It might be fruitful to explore the possible different impact of ACL algorithms in BCIs based on the mentioned distinct physiological mechanisms, especially with regard to their specific BCI illiteracies.

As mentioned in section “Baseline Resting State EEG Measures as Possible Interindividual Correlates of ITR Performances,” the IAF is a measure of *interindividual* EEG variability because it is remarkably stable *within* subjects, but relatively variable *between* subjects (Kondacs and Szabó, 1999). IAF seems to be highly heritable, e.g., Posthuma et al. (2001) found in a study comparing mono- vs. dizygotic twins, analyzing a large representative sample of healthy Dutch adults (*N* = 688), that 71–83% of total IAF variance could be ascribed to genetic variances. Thus, IAF may be considered as an *endophenotype* following the definition of Gottesman and Gould (2003). Klimesch (1997) found in a sample of age matched subjects that the IAF of good working memory performers is about 1 Hz higher vs. that of bad performers. Jin et al. (2006) found that IAF is positively correlated with conflict reaction time. Severity of *Alzheimer's* disease is positively related to the extent of typical IAF slowing in this pathology (Rodriguez et al., 1999). On the neurophysiological level, Steriade et al. (1990) reported that IAF depends on membrane properties of the thalamic neurons which project to the cortex, implying thalamo-cortical feedback loops as one of the important generators of alpha activity (Lopes da Silva, 1991). Mayer et al. (2007) successfully modeled the synchronization of locally coupled bistable thalamic oscillators as controlled by the influence of corticothalamic projections, probably responsible for widespread spindle oscillations in the thalamus. Given these findings, IAF might be understood as a positive correlate of thalamo-cortical information processing speed. With regard of possible correlations of IAF with SSVEP magnitudes, Koch et al. (2008) found interesting correlations of IAF with both magnitudes of VEPs and cortical oxygenation measured by NIRS. Concluding, IAF seems to open new insights into the understanding of the neural circuits underlying BCI performance and thus should be considered as a promising predictor for further studies.

In this study, only eight EEG electrodes were used to investigate EEG resting state measures as performance predictors, but further works might use more electrodes of the 10–20 system to allow a-posteriori offline analyses of *scalp maps* and the use of *source localization* techniques, e.g., *LORETA* (for a review see Grech et al., 2008). Findings of research concerning the cerebral *resting-state networks* call for further studies which use simultaneous EEG/fMRI recordings (for reviews see e.g., Fox and Raichle, 2007; Van den Heuvel and Hulshoff-Pol, 2010; for typical studies see e.g., Damoiseaux et al., 2006; Van den Heuvel et al., 2009; Yuan et al., 2012).

Opening the scope to other uses, the demonstrated advantage of our adaptive-interactive BCI protocol can be expanded conceptually, e.g., to innovative applications such as diagnostic/therapeutic tools in clinical contexts: exploring the subject-specific dynamical trajectory of machine-subject interaction could extract information which otherwise would remain undiscovered. Thus, far beyond an engineering focus, the proposed approach might be employed as a new paradigm for basic neuroscientific and biomedical research.

### Conflict of interest statement

The authors declare that the research was conducted in the absence of any commercial or financial relationships that could be construed as a potential conflict of interest.
